# Seasonal and pandemic influenza during pregnancy and risk of fetal death: A Norwegian registry-based cohort study

**DOI:** 10.1007/s10654-020-00600-z

**Published:** 2020-01-16

**Authors:** Nina Gunnes, Håkon Kristian Gjessing, Inger Johanne Bakken, Sara Ghaderi, Jon Michael Gran, Olav Hungnes, Per Magnus, Sven Ove Samuelsen, Anders Skrondal, Camilla Stoltenberg, Lill Trogstad, Allen J. Wilcox, Siri Eldevik Håberg

**Affiliations:** 1 Norwegian Institute of Public Health, Oslo, Norway; 2 Norwegian National Advisory Unit on Women’s Health, Oslo University Hospital, Oslo, Norway; 3 Centre for Fertility and Health, Norwegian Institute of Public Health, Oslo, Norway; 4 Department of Global Public Health and Primary Care, University of Bergen, Bergen, Norway; 5 Oslo Centre for Biostatistics and Epidemiology, University of Oslo and Oslo University Hospital, Oslo, Norway; 6 Department of Mathematics, University of Oslo, Oslo, Norway; 7 University of California, Berkeley, CA USA; 8 Centre for Educational Measurement, University of Oslo, Oslo, Norway; 9 National Institute of Environmental Health Sciences, Research Triangle Park, NC USA

**Keywords:** Fetal death, Influenza-like illness, Miscarriage, Pandemic influenza, Pregnancy, Seasonal influenza, Spontaneous abortion, Stillbirth

## Abstract

Previous studies of fetal death with maternal influenza have been inconsistent. We explored the effect of maternal influenza-like illness (ILI) in pregnancy on the risk of fetal death, distinguishing between diagnoses during regular influenza seasons and the 2009/2010 pandemic and between trimesters of ILI. We used birth records from the Medical Birth Registry of Norway to identify fetal deaths after the first trimester in singleton pregnancies (2006–2013). The Norwegian Directorate of Health provided dates of clinical influenza diagnoses by primary-health-care providers, whereas dates of laboratory-confirmed influenza A (H1N1) diagnoses were provided by the Norwegian Surveillance System for Communicable Diseases. We obtained dates and types of influenza vaccinations from the Norwegian Immunisation Registry. Cox proportional-hazards regression models were fitted to estimate hazard ratios (HRs) of fetal death, with associated 95% confidence intervals (CIs), comparing women with and without an ILI diagnosis in pregnancy. There were 2510 fetal deaths among 417,406 eligible pregnancies. ILI during regular seasons was not associated with increased risk of fetal death: adjusted HR = 0.90 (95% CI 0.64–1.27). In contrast, ILI during the pandemic was associated with substantially increased risk of fetal death, with an adjusted HR of 1.75 (95% CI 1.21–2.54). The risk was highest following first-trimester ILI (adjusted HR = 2.28 [95% CI 1.45–3.59]). ILI during the pandemic—but not during regular seasons—was associated with increased risk of fetal death in the second and third trimester. The estimated effect was strongest with ILI in first trimester.

**Electronic supplementary material:**

The online version of this article (10.1007/s10654-020-00600-z) contains supplementary material, which is available to authorized users.

## Introduction

Several previous studies have assessed associations between maternal influenza virus infection in pregnancy and adverse maternal and pregnancy outcomes [1, 2].

Pregnant women are at increased risk of morbidity related to seasonal influenza [3–5]. However, there is conflicting evidence of associations with perinatal outcomes, as findings are inconsistent across studies [6–10]. A US study of respiratory illness in pregnant women found no statistically significant association between respiratory hospitalization during influenza seasons in 1985–1993 and fetal death [7]. In a Danish study of the temporal association between influenza activity and frequency of fetal death in 1994–2009, there was no statistically significant correlation between time series of weekly influenza-like illness (ILI) consultation proportions and time series of weekly proportions of spontaneous abortions or stillbirths [11]. On the other hand, a US study of pregnant women hospitalized during influenza seasons in 1998–2008 demonstrated increased odds of intrauterine fetal demise among delivery hospitalizations complicated by respiratory illness compared to non-respiratory delivery hospitalizations [12]. However, this study was considered to be at a very high risk of diagnostic ascertainment bias [2].

Maternal morbidity and adverse pregnancy and neonatal outcomes have been associated with 2009 pandemic influenza A virus subtype H1N1 (influenza A[H1N1]pdm09) infection in pregnancy [13–15]. A UK study reported an association between maternal influenza A(H1N1)pdm09 infection in pregnancy and increased risk of fetal death from 24 weeks [16], but the risk of diagnostic ascertainment bias for this study has been considered high [2]. In a Chinese study of maternal influenza A(H1N1)pdm09 infection in pregnancy, stillbirth was more frequent among women who tested positive for virus-specific antibodies compared to women who tested negative [17]. Furthermore, the risk of fetal death was increased following a primary-health-care diagnosis of influenza in pregnancy during the “swine flu” pandemic in 2009/2010 in Norway [18].

The main objective of the current study was to assess the risk of fetal death associated with maternal ILI in pregnancy during the influenza seasons between 2006 and 2013. In addition, we aimed to explore the associations by trimester of ILI.

## Methods

We used individual-level data from Norwegian nationwide health registries. The data sources were linked by the unique personal identification number assigned to every resident of Norway.

### Fetal death

The Medical Birth Registry of Norway (MBRN) contains data on births after 12 completed weeks of pregnancy, including detailed information about the mother, the pregnancy, the delivery, and the child. Fetal death was defined as any fetal loss, i.e., spontaneous abortion (miscarriage) or stillbirth, from the 12th completed gestational week onwards. We used the MBRN to identify fetal deaths in the second and third trimester in the years 2006–2013.

### Influenza seasons

The Norwegian Institute of Public Health (NIPH) is responsible for monitoring the influenza activity in Norway throughout the whole year [19, 20]. However, the surveillance of medical consultations in primary health care related to ILI is restricted from calendar week 40 (end of September or beginning of October) to calendar week 20 (middle of May). We considered nine different influenza seasons from 2006 (including the 2005/2006 season) through 2013 (including the 2013/2014 season). All seasons coincided with the NIPH’s annual surveillance of influenza-related medical consultations but the pandemic season in 2009/2010, which we defined from mid-May 2009 to mid-May 2010.

### Influenza-like illness

In Norway, all consultation dates and diagnoses made by a general practitioner in primary health care are reported to the Norwegian Directorate of Health for reimbursement purposes [21]. Diagnoses are coded using the International Classification of Primary Care, Second Edition (ICPC-2) [22]. We obtained data on all women diagnosed with ICPC-2 code R80 (“influenza”) in 2006–2013. Furthermore, data on women with a laboratory-confirmed diagnosis of influenza A (H1N1) virus infection, i.e., “swine flu”, in 2009–2011 were obtained from the Norwegian Surveillance System for Communicable Diseases (MSIS).

The R80 diagnosis from primary health care is based on several clinical criteria of influenza symptoms occurring within an influenza outbreak. Criteria for the R80 code are given in Online Resource 1. We only included R80 diagnoses made during the predefined influenza seasons. Thus, we discarded all “off-season” R80 diagnoses. Laboratory-confirmed influenza A (H1N1) diagnoses were, however, considered reliable regardless of whether they were given within or outside the influenza seasons in question; all laboratory-confirmed diagnoses before July 1, 2010, were attributed to the 2009/2010 pandemic season (first observed diagnosis on June 10, 2009), and all laboratory-confirmed diagnoses from July 1, 2010, onwards were attributed to the subsequent season in 2010/2011 (last observed diagnosis on April 5, 2011). We considered ILI as either an R80 diagnosis or a laboratory-confirmed influenza A (H1N1) diagnosis.

Following the 2009/2010 pandemic season, influenza A(H1N1)pdm09 was regarded as one of the seasonal influenza strains, and infection with this virus subtype variant was thereafter considered as regular seasonal influenza. Hence, we defined *pandemic influenza* as ILI related to the 2009/2010 season and *seasonal influenza* as ILI related to any of the other eight influenza seasons under study, regardless of the circulating strains.

### Influenza vaccination

Data on vaccination against influenza virus were provided by the Norwegian Immunisation Registry (SYSVAK) from 2006 through the beginning of 2013 [23]. Influenza vaccinations were not notifiable prior to the “swine flu” pandemic in 2009/2010, during which reporting of all influenza vaccinations to SYSVAK was mandatory and nearly complete. After the pandemic, that is, from the 2010/2011 season onwards, influenza vaccinations have been notifiable provided that oral consent is obtained from each vaccinee or parent/guardian [23]. Hence, there has been a considerable underreporting of influenza vaccinations during regular seasons. Therefore, we only considered vaccinations with Pandemrix H1N1^®^ (GlaxoSmithKline) and Celvapan^®^ (Baxter), which were vaccines used during the pandemic influenza outbreak. The proportion between the two vaccination types in the current study was 2.2 Celvapan^®^ vaccinations per 10 000 Pandemrix H1N1^®^ vaccinations. These will hereafter be referred to as *pandemic vaccinations*.

### Study sample

The MBRN data set (2006–2013) initially comprised 486,008 birth records among 336,877 mothers. Birth records corresponding to induced abortions were not included. We discarded 3527 records with missing data on pregnancy onset due to missing length of gestation and 46,022 records with pregnancy onset before January 1, 2006, that is, before information about ILI diagnoses was available. We also discarded 3321 records with pregnancy onset after March 5, 2013, to make sure that all pregnancies under study had the potential of lasting 43 completed weeks (301 days), thereby avoiding an oversampling of short pregnancies towards the end of 2013 due to right-truncation [18]. We further discarded 9 records with missing or invalid maternal residential status as registered in the National Registry and 471 records corresponding to maternal emigration before pregnancy onset or January 1, 2006. Moreover, we discarded 933 records of live-born children born to foreign parents not living in Norway and 158 records registered as live births but with gestational length less than 22 weeks or birth weight less than 500 g. In addition, we discarded 16,774 records with plurality greater than one or missing. Several birth records were discarded for more than one of the aforementioned reasons. Finally, our study sample comprised 417,406 eligible singleton (i.e., non-plural) birth records (305,180 mothers), of which 2510 records (0.6%) of fetal death. Characteristics of the study sample are shown in Table 1.

**Table 1:** Characteristics of the study sample from the Medical Birth Registry of Norway (2006–2013)

	Live births		Fetal deaths^a^		Total number
	Number	Percentage	Number	Percentage	
Total	414,896	99.4	2510	0.6	417,406
Year of birth					
2006^b^	12,011	98.4	191	1.6	12,202
2007	56,027	99.3	377	0.7	56,404
2008	58,128	99.4	372	0.6	58,500
2009	59,487	99.5	320	0.5	59,807
2010	59,122	99.4	345	0.6	59,467
2011	58,122	99.4	340	0.6	58,462
2012	58,109	99.4	325	0.6	58,434
2013^c^	53,890	99.6	240	0.4	54,130
Gestational length (completed weeks)					
12–19	0	0.0	714	100.0	714
20–27	901	54.6	748	45.4	1649
28–35	11,470	96.6	398	3.4	11,868
36–43	402,350	99.8	650	0.2	403,000
≥ 44	175	100.0	0	0.0	175
Maternal age (years)					
< 20	8645	99.3	65	0.7	8710
20–24	60,702	99.5	310	0.5	61,012
25–29	130,208	99.5	618	0.5	130,826
30–34	135,832	99.4	795	0.6	136,627
35–39	66,677	99.1	587	0.9	67,264
≥ 40	12,832	99.0	135	1.0	12,967
Maternal marital status					
Married/co-habitant	382,604	99.4	2209	0.6	384,813
Other	32,292	99.1	301	0.9	32,593
Maternal parity					
0	175,831	99.5	961	0.5	176,792
≥ 1	239,065	99.4	1549	0.6	240,614
Maternal history of fetal death					
No	325,860	99.5	1734	0.5	327,594
Yes	89,036	99.1	776	0.9	89,812
Maternal chronic conditions^d^					
No	366,145	99.4	2211	0.6	368,356
Yes	48,751	99.4	299	0.6	49,050
Maternal use of nutritional supplements before and/or during pregnancy					
No	111,167	98.9	1256	1.1	112,423
Yes	303,729	99.6	1254	0.4	304,983
Maternal smoking at the beginning of pregnancy					
No	311,389	99.4	1775	0.6	313,164
Yes	103,507	99.3	735	0.7	104,242
Maternal seasonal influenza during pregnancy					
No	406,588	99.4	2477	0.6	409,065
Yes	8308	99.6	33	0.4	8341
Trimester of maternal seasonal influenza during pregnancy					
No seasonal influenza during pregnancy	406,588	99.4	2477	0.6	409,065
First trimester	3031	99.3	20	0.7	3051
Second or third trimester	5277	99.8	13	0.2	5290
Maternal pandemic influenza during pregnancy					
No	411,343	99.4	2482	0.6	413,825
Yes	3553	99.2	28	0.8	3581
Trimester of maternal pandemic influenza during pregnancy					
No pandemic influenza during pregnancy	411,343	99.4	2482	0.6	413,825
First trimester	1387	98.6	19	1.4	1406
Second or third trimester	2166	99.6	9	0.4	2175

^a^Spontaneous abortions (miscarriages) and stillbirths

^b^Excluding pregnancies with onset before January 1, 2006

^c^Excluding pregnancies with onset after March 5, 2013

^d^Asthma, chronic hypertension, chronic renal disease, rheumatoid arthritis, heart disease, epilepsy, diabetes, or thyroid disease

### Statistical analysis

We applied Cox proportional-hazards regression to estimate crude and adjusted hazard ratios (HRs) of fetal death between different groups: women diagnosed with ILI at various stages of pregnancy (any time in pregnancy, in the first trimester, and in the second or third trimester, respectively) and undiagnosed women. Associated 95% confidence intervals (CIs) were obtained by using robust standard errors, allowing for intragroup correlation between birth records with shared mother (i.e., siblings).

Our time scale was the number of days since pregnancy onset, which was established by using information from the MBRN about date of birth and gestational length. The latter was based on estimations from an ultrasound scan, if available; otherwise, it was based on the first day of the last menstrual period, as reported by the mother. The event of interest was fetal death. We defined censoring as either a live birth, maternal emigration, or pregnancy day 301 (first day of the 43rd completed gestational week). The study period extended from January 1, 2006, to December 31, 2013, during which data on both ILI and fetal death were available. As the study sample only included birth records with pregnancy onset up to, and including, March 5, 2013, no observations were censored due to end of study (December 31, 2013).

The exposures under study were seasonal and pandemic influenza during pregnancy. A woman was regarded as being exposed to a specific influenza type from the earliest date of an ILI diagnosis in pregnancy during a relevant season, designated as the type-specific ILI onset date, throughout the rest of her pregnancy. We fitted two different regression models. Model 1 included two indicator variables for having had seasonal/pandemic influenza at any time during pregnancy: ‘no’ or ‘yes’. In Model 2, we included two categorical variables for seasonal/pandemic influenza during pregnancy, incorporating the timing of the respective exposures: ‘no’, ‘yes, in the first trimester’, or ‘yes, in the second or third trimester’. The influenza exposures were handled as time-dependent variables, treating women as unexposed until the first pregnancy day of an ILI diagnosis.

To explore how the effects of seasonal and pandemic influenza might vary with time during the course of pregnancy, we conducted three separate sets of analyses of fetal death at different gestational stages. The main analyses considered fetal death at any time after the first trimester. In these analyses, women entered the risk set on pregnancy day 84 (first day of the 12th completed gestational week) and were followed until either fetal death or censoring, whichever occurred first. In a set of supplementary analyses, we studied fetal death in the second trimester only. As in the main analyses, we followed the women from pregnancy day 84. Observations of women neither delivering nor emigrating during the second trimester were censored on pregnancy day 195 (last day of the 27th completed gestational week). Another set of supplementary analyses were restricted to fetal death in the third trimester. Here, women first entered the risk set on pregnancy day 196 (first day of the 28th completed gestational week). The last possible exit time in these analyses was on pregnancy day 301 (first day of the 43rd completed gestational week), as in the main analyses.

In some additional supplementary analyses, we also adjusted for pandemic vaccination. The study period for these analyses was restricted from May 15, 2009, to September 30, 2010, thereby including only the pandemic season and none of the regular influenza seasons. We fitted two regression models, Model 3 and Model 4 (analogous to Model 1 and Model 2, respectively), including exposure variables for pandemic influenza and pandemic vaccination. Seasonal influenza was not included in these analyses.

We obtained information on seven variables from the MBRN based on a priori considerations of potential confounders of the associations between maternal seasonal and pandemic influenza during pregnancy and fetal death. These included maternal age (‘< 20 years’, ‘20–24 years’, ‘25–29 years’, ‘30–34 years’, ‘35–39 years’, or ‘≥ 40 years’), maternal marital status (‘married/co-habitant’ or ‘other’), maternal parity (‘0’ or ‘≥ 1’), maternal history of fetal death (‘no’ or ‘yes’), maternal chronic conditions such as asthma, chronic hypertension, chronic renal disease, rheumatoid arthritis, heart disease, epilepsy, diabetes, and thyroid disease (‘no’ or ‘yes’), maternal use of nutritional supplements before and/or during pregnancy (‘no’ or ‘yes’), and maternal smoking at the beginning of pregnancy (‘no’ or ‘yes’). The variables were all included in the adjusted analyses as baseline covariates. In addition, we assumed that the current calendar quarter (‘January through March’, ‘April through June’, ‘July through September’, or ‘October through December’) might possibly also affect both the pregnant woman’s influenza status and the risk of fetal death. Thus, calendar quarter was included as a time-dependent covariate in the adjusted analyses to account for seasonal variation in the exposures and outcome.

Data were analyzed by using Stata SE 15 (StataCorp. 2017. Stata Statistical Software: Release 15. College Station, TX: StataCorp LLC).

## Results

There were 2510 fetal deaths among the 417,406 eligible singleton birth records (6.0/1000). A total of 11,896 birth records (2.8%) were linked to maternal influenza during pregnancy: 8341 records (2.0%) to seasonal influenza and 3581 records (0.9%) to pandemic influenza (Table 1). A few birth records (26, < 0.1%) were linked to both seasonal and pandemic influenza. There was a marked predominance of clinical R80 diagnoses from primary health care over laboratory-confirmed H1N1 diagnoses among the ILI cases. During regular influenza seasons, 8327 birth records were registered with at least one maternal R80 diagnosis in pregnancy, compared to 41 birth records with at least one maternal laboratory-confirmed H1N1 diagnosis in pregnancy. Corresponding numbers for the pandemic season were 3479 and 518, respectively. There were too few fetal deaths among those with a laboratory-confirmed H1N1 diagnosis in pregnancy to conduct any sensitivity analyses restricted to such diagnoses.

The crude fetal mortality was 4.0 (95% CI 2.6–5.3) per 1000 total births (i.e., live births and fetal deaths) among women diagnosed with seasonal influenza during pregnancy and 6.1 (95% CI 5.8–6.3) per 1000 total births among women *not* diagnosed with seasonal influenza. For pandemic influenza, the crude fetal mortality was 7.8 (95% CI 4.9–10.7) and 6.0 (95% CI 5.8–6.2) per 1000 total births among diagnosed and undiagnosed women, respectively.

**Fig. 1:**
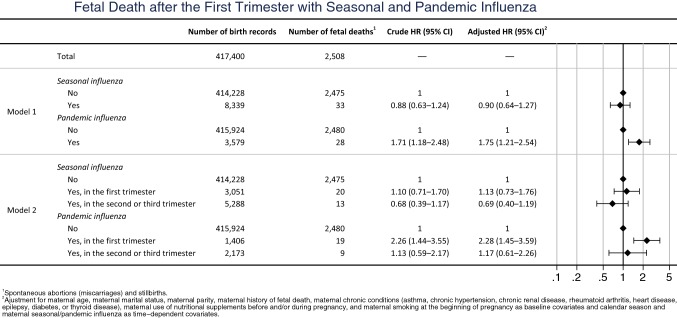
Hazard ratios (HRs) of fetal death in the second or third trimester, with associated 95% confidence intervals (CIs), between women with and without a diagnosis of influenza-like illness in pregnancy during regular influenza seasons (seasonal influenza) and with and without a diagnosis of influenza-like illness in pregnancy during the 2009/2010 pandemic season (pandemic influenza), respectively. Estimated by using Cox proportional-hazards regression with follow-up between January 1, 2006, and December 31, 2013. Adjusted HRs also displayed graphically to the far right

Results from the main analyses are displayed in Fig. 1. We found no evidence that maternal seasonal influenza during pregnancy was associated with increased risk of fetal death after the first trimester, either overall (adjusted HR: 0.90 [95% CI 0.64–1.27]) (Model 1) or by trimester of first ILI diagnosis during a regular influenza season (first-trimester adjusted HR: 1.13 [95% CI 0.73–1.76]; second- or third-trimester adjusted HR: 0.69 [95% CI 0.40–1.19]) (Model 2). On the other hand, the risk of fetal death was higher following maternal pandemic influenza during pregnancy (adjusted HR: 1.75 [95% CI 1.21–2.54]) (Model 1). This association seemed to be stronger with first ILI diagnosis during the pandemic season in the first trimester (adjusted HR: 2.28 [95% CI 1.45–3.59]) than in the second or third trimester (adjusted HR: 1.17 [95% CI 0.61–2.26]) (Model 2).

Results from supplementary analyses when restricting to fetal deaths in the second and third trimester, respectively, were similar to the main results (Supplementary Figs. 1 and 2, Online Resource 2).

In the supplementary analyses restricted to the pandemic season in 2009/2010, the confounding effect of pandemic vaccination during pregnancy on the association between pandemic influenza during pregnancy and risk of fetal death was deemed negligible. Adjusting for pandemic vaccination only slightly changed the effect of pandemic influenza on the risk of fetal death—both overall (Model 3) and when considering the respective trimesters of first ILI diagnosis during the pandemic season and vaccination (Model 4) (Supplementary Figs. 3–5, Online Resource 2).

The proportional-hazards assumption of the influenza exposures under study was tested on the basis of Schoenfeld residuals by using the $\tt{estat \,phtest}$ command in Stata after fitting the adjusted regression models. This involves testing that the log of the hazard ratio is constant over time. There was no evidence that the proportional-hazards assumption had been violated for any of the influenza variables in the adjusted main analyses; all corresponding *p* values in Model 1 and Model 2 exceeded 0.05—both for seasonal influenza (overall: 0.46; first trimester: 0.13; second or third trimester: 0.19) and for pandemic influenza (overall: 0.13; first trimester: 0.47; second or third trimester: 0.39).

## Discussion

Maternal pandemic influenza during pregnancy—especially in the first trimester—was associated with a substantial increase in risk of fetal death later in pregnancy. Our results suggest that the risk of fetal death following ILI during the pandemic season remained elevated throughout the entire pregnancy.

The literature is inconsistent with regard to an effect of influenza on fetal mortality during previous pandemics. A US study of influenza in pregnant women during the “Spanish flu” pandemic of 1918/1919 showed that interruption of pregnancy (spontaneous abortion or preterm birth) was 52% among cases in which influenza was complicated by pneumonia, compared to 26% among uncomplicated influenza cases [24]. In a study of pregnant women in England during the “Asian flu” pandemic of 1957, there was no clear evidence of increased stillbirths following influenza in pregnancy [25]. A US study of “Asian flu” also found no increase in stillbirths [26]. No statistically significant difference in the incidence of fetal deaths between infected and uninfected women was found in a US study of teratogenic effects of pandemic “Asian flu” during pregnancy [27]. However, in another US study of the same outbreak, spontaneous abortions and stillbirths were more frequent in women infected during their first trimester than those afflicted later in pregnancy or in uninfected women [28].

In contrast with our results for pandemic influenza, we found no evidence that maternal ILI during regular seasonal influenza increased the risk of fetal death. There are several possible explanations. The effects of infection may differ by the strain of influenza. It would have been of interest to separate strains, but, unfortunately, such information was not available in the current study. However, this explanation seems unlikely in that influenza virus from the H1N1 strain was circulating during several of the regular influenza seasons following the “swine flu” pandemic of 2009/2010 [29].

Another possible explanation is that pandemic influenza virus infections may be more severe, perhaps due to reduced immunity in the population against the new (pandemic) viruses. In Norway, the prevalence of preexisting protective antibodies to the 2009 pandemic influenza A(H1N1) virus (hemagglutination inhibition [HI] titer ≥ 40) among adults aged 20–29 years and 30–49 years in August 2008 were only 3.9% and 1.3%, respectively [30].

The current study extends a previous Norwegian study based on data from the same registries and databases but restricted to pregnancies during the pandemic influenza outbreak of 2009/2010 [18]. By extending the study period, we were able to include non-pandemic influenza seasons as well. We also considered trimester-specific effects of ILI. The previous study found pandemic influenza in pregnancy to be associated with increased risk of fetal death, whereas there was no apparent effect of pandemic vaccination on the risk of fetal death [18]. In the current study, the risk of fetal death appeared to be somewhat higher with ILI in the first trimester during the pandemic season.

A major strength of our study was the use of individual-level data from independent, mandatory registries with nationwide coverage and high data quality. For instance, the validity of diagnoses of unexplained antepartum fetal death as registered in the MBRN has previously been found to be sufficiently high for large-scale epidemiologic studies [31]. Large population-based registries are crucial to the surveillance of a variety of exposures and outcomes, particularly when clinical trials are not possible. Moreover, many small-sample studies are hampered by low statistical power—especially in the case of rare exposures or outcomes. This is less of a concern in large registry-based studies.

Another important strength of the current study was that all analyses were conducted by using Cox proportional-hazards regression with time-dependent exposure variables. By choosing a time-to-event approach, we were able to take the time aspect of the exposure (influenza during pregnancy) and the outcome (fetal death) into account, thereby avoiding potential immortal time bias (also known as time-dependent bias), where time at risk of outcome before exposure is misclassified as being associated with the exposure [32]. Furthermore, we could explore whether the effects of the trimester-specific influenza exposures differed across trimesters based on separate analyses of fetal death restricted to the second and third trimester, respectively.

A limitation of our study was uncertainty related to the accuracy of influenza diagnoses from primary health care. More precisely, we lacked confirmation that the R80 diagnoses reflected true influenza infections, as they are based mostly on clinical symptoms and not routinely laboratory-confirmed. However, other ICPC-2 codes for unspecific symptoms such as “upper respiratory infection, acute” (R74), “cough” (R05), and “fever” (A03) are much more commonly used in primary health care, suggesting that a code for influenza is based on a more thorough evaluation of the clinical picture. Although only a small fraction of patients had laboratory confirmation of influenza, primary-health-care physicians are instructed to consider whether there is influenza in close contacts or an ongoing influenza epidemic, in addition to other relevant criteria for influenza, before assigning a diagnosis (Online Resource 1). Even so, some R80 diagnoses may be a result of similar symptoms caused by other respiratory viruses (i.e., differential diagnoses of influenza virus infection). To increase the reliability of influenza infections, we therefore discarded all R80 diagnoses in pregnancy made outside the predefined influenza seasons under study. This reduced the number of birth records in the study sample with at least one influenza diagnosis in pregnancy by 6.5%: from 12,728 to 11,896 birth records. Moreover, all influenza diagnoses were limited to women seeking medical care, and with that, we no doubt misclassified cases of asymptomatic/subclinical or mild, less severe infections of influenza as unexposed. Hence, we expect the current study to reflect associations with more severe influenza.

Our study lacked information about fetal losses in the first trimester, which made it impossible to address acute effects of maternal ILI occurring early in pregnancy. However, as we found increased risk of later fetal death with pandemic influenza in the first trimester, there is a possibility that this effect also increased losses in the first trimester. If so, we have underestimated the overall risk of fetal death associated with first-trimester ILI during the pandemic season.

An additional limitation of our study was not being able to make a proper adjustment for influenza vaccination due to the lack of complete information on all vaccinations against influenza virus. During the pandemic, about 90% of all vaccinees receiving at least one dose of vaccine against 2009 pandemic influenza were registered in SYSVAK [23]. The estimated annual seasonal influenza vaccine coverage in the general Norwegian population in 2006–2017 was approximately 10% (unpublished data based on distributed doses, unused doses, and vaccinations registered in SYSVAK). Uptake is likely to be higher in defined risk groups. It is conceivable that vaccination might affect the risk of fetal death while simultaneously reducing the risk of ILI in the pregnant woman, thereby confounding the association between maternal influenza during pregnancy and risk of fetal death. However, the low completeness of seasonal influenza vaccinations reported to SYSVAK limited our ability to include these in our study. Pandemic vaccination has previously been shown not to be associated with increased risk of fetal death [18]. Accordingly, adjustment for pandemic vaccination in the current study had little impact on the estimates for pandemic influenza in the supplementary analyses restricted to mid-May 2009 through September 2010, indicating that pandemic vaccination may not be an important confounder in this respect.

The MBRN requires registration of pregnancy losses at 12 completed weeks or more. Fetal deaths before that time could not be included in our study. Even starting at 12 weeks, pregnancy losses are probably under-ascertained in the earliest weeks of the second trimester. Thus, some early second-trimester fetal deaths are likely to be missing from our study sample. If registrations of early pregnancy losses from birth clinics were dependent on the mothers being diagnosed with influenza in primary health care, our results could be inflated. However, the registries are managed independently, and the increased risk after infection was present throughout pregnancy, making this potential bias less of a concern.

This study demonstrates the strengths and importance of nationwide population-based registries. It also indicates that such databases must be improved and expanded in order to fully meet the needs of public-health surveillance.

Future studies should attempt to address the biological and pathogenic mechanisms underlying the relation between maternal influenza virus infection in pregnancy and fetal death, with pandemic influenza virus infection being of special interest. Such mechanisms might include transplacental transmission of the virus as well as transmission of inflammation products and maternal antibodies. There is also a need to explore the possibility of fetal losses in the first trimester with first-trimester infection.

In conclusion, there was no evidence of any increase in the risk of fetal death in the second or third trimester with maternal seasonal influenza. However, we found a more than twofold increased risk of fetal death following maternal pandemic influenza in the first trimester, supporting that influenza during the pandemic season in 2009/2010 was a more severe threat to the fetus than influenza during regular seasons.

## Electronic supplementary material

Below is the link to the electronic supplementary material.

Supplementary material 1 (PDF 501 kb)

Supplementary material 2 (PDF 1353 kb)
