# Supplementary material for: Seasonal and pandemic influenza during pregnancy and risk of fetal death: A Norwegian registry-based cohort study
Source: Eur J Epidemiol. 2020 Jan 16;35(4):371–9. doi: 10.1007/s10654-020-00600-z (PMC7192880; doi:10.1007/s10654-020-00600-z)
Supplement: Supplementary file 1 — Supplementary material 1 (PDF 501 kb) [file 10654_2020_600_MOESM1_ESM.pdf]

Journal: European Journal of Epidemiology

Title: Seasonal and pandemic influenza during pregnancy and risk of fetal death: A Norwegian registry-based cohort study

Authors: Nina Gunnes[1,2], Håkon Kristian Gjessing[3,4], Inger Johanne Bakken[3], Sara Ghaderi[4], Jon Michael Gran[5], Olav Hungnes[1], Per Magnus[3], Sven Ove Samuelsen[1,6], Anders Skrondal[3,7,8], Camilla Stoltenberg[1,4], Lill Trogstad[1], Allen J Wilcox[9], Siri Eldevik Håberg[3]

Affiliations: [1]Norwegian Institute of Public Health, Oslo, Norway; [2]Norwegian National Advisory Unit on Women's Health, Oslo University Hospital, Oslo, Norway; [3]Centre for Fertility and Health, Norwegian Institute of Public Health, Oslo, Norway; [4]Department of Global Public Health and Primary Care, University of Bergen, Bergen, Norway; [5]Oslo Centre for Biostatistics and Epidemiology, University of Oslo and Oslo University Hospital, Oslo, Norway; [6]Department of Mathematics, University of Oslo, Oslo, Norway; [7]University of California, Berkeley, CA, USA; [8]Centre for Educational Measurement, University of Oslo, Oslo, Norway; [9]National Institute of Environmental Health Sciences, Research Triangle Park, NC, USA

Corresponding author: Nina Gunnes (Norwegian National Advisory Unit on Women's Health, Oslo University Hospital, Oslo, Norway; ninagu@ous-hf.no)

Influenza diagnosis

Women in the current study were diagnosed with influenza using the International Classification of Primary Care, Second Edition (ICPC-2) code R80.

ICPC-2 Code R80

Inclusion: influenza-like illness; para-influenza

Exclusion: gastric flu D70; influenza pneumonia R81

Criteria: myalgia and cough without abnormal respiratory physical signs other than inflammation of nasal mucous membrane and throat, plus three or more of the following: sudden onset (within 12 hours); rigors/chills/fever; prostration and weakness; influenza in close contacts; influenza epidemic; or viral culture/serological evidence of influenza virus infection

Consider: fever A03; virus infection NOS A77; upper respiratory tract infection R74
