# Supplementary material for: Seasonal and pandemic influenza during pregnancy and risk of fetal death: A Norwegian registry-based cohort study
Source: Eur J Epidemiol. 2020 Jan 16;35(4):371–9. doi: 10.1007/s10654-020-00600-z (PMC7192880; doi:10.1007/s10654-020-00600-z)
Supplement: Supplementary file 2 — Supplementary material 2 (PDF 1353 kb) [file 10654_2020_600_MOESM2_ESM.pdf]

Journal: European Journal of Epidemiology

Title: Seasonal and pandemic influenza during pregnancy and risk of fetal death: A Norwegian registry-based cohort study

Authors: Nina Gunnes[1,2], Håkon Kristian Gjessing[3,4], Inger Johanne Bakken[3], Sara Ghaderi[4], Jon Michael Gran[5], Olav Hungnes[1], Per Magnus[3], Sven Ove Samuelsen[1,6], Anders Skrondal[3,7,8], Camilla Stoltenberg[1,4], Lill Trogstad[1], Allen J Wilcox[9], Siri Eldevik Håberg[3]

Affiliations: [1]Norwegian Institute of Public Health, Oslo, Norway; [2]Norwegian National Advisory Unit on Women's Health, Oslo University Hospital, Oslo, Norway; [3]Centre for Fertility and Health, Norwegian Institute of Public Health, Oslo, Norway; [4]Department of Global Public Health and Primary Care, University of Bergen, Bergen, Norway; [5]Oslo Centre for Biostatistics and Epidemiology, University of Oslo and Oslo University Hospital, Oslo, Norway; [6]Department of Mathematics, University of Oslo, Oslo, Norway; [7]University of California, Berkeley, CA, USA; [8]Centre for Educational Measurement, University of Oslo, Oslo, Norway; [9]National Institute of Environmental Health Sciences, Research Triangle Park, NC, USA

Corresponding author: Nina Gunnes (Norwegian National Advisory Unit on Women's Health, Oslo University Hospital, Oslo, Norway; ninagu@ous-hf.no)

Fetal Death in the Second Trimester with Seasonal and Pandemic Influenza

		Number of birth records	Number of fetal deaths[1]	Crude HR (95% CI)	Adjusted HR (95% CI)[2]
	Total	417,400	1,460	—	—
Model 1	*Seasonal influenza*				
	No	414,228	1,444	1	1
	Yes	6,621	16	0.91 (0.56–1.49)	0.93 (0.57–1.52)
	Pandemic influenza				
	No	415,924	1,444	1	1
	Yes	2,870	16	2.03 (1.24–3.32)	2.11 (1.29–3.46)
Model 2	*Seasonal influenza*				
	No	414,228	1,444	1	1
	Yes, in the first trimester	3,051	13	1.22 (0.71–2.10)	1.24 (0.72–2.14)
	Yes, in the second trimester	3,570	3	0.44 (0.14–1.35)	0.45 (0.14–1.39)
	Pandemic influenza				
	No	415,924	1,444	1	1
	Yes, in the first trimester	1,406	11	2.26 (1.25–4.08)	2.28 (1.26–4.13)
	Yes, in the second trimester	1,464	5	1.65 (0.69–3.98)	1.80 (0.75–4.33)

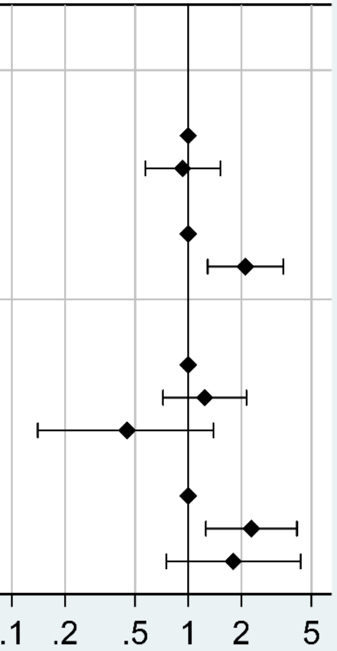

[1]Spontaneous abortions (miscarriages) and stillbirths.
[2]Ajustment for maternal age, maternal marital status, maternal parity, maternal history of fetal death, maternal chronic conditions (asthma, chronic hypertension, chronic renal disease, rheumatoid arthritis, heart disease, epilepsy, diabetes, or thyroid disease), maternal use of nutritional supplements before and/or during pregnancy, and maternal smoking at the beginning of pregnancy as baseline covariates and calendar season and maternal seasonal/pandemic influenza as time-dependent covariates.

Supplementary Fig. 1 *Hazard ratios (HRs) of fetal death in the second trimester, with associated 95% confidence intervals (CIs), between women with and without a diagnosis of influenza-like illness in pregnancy during regular influenza seasons (seasonal influenza) and with and without a diagnosis of influenza-like illness in pregnancy during the 2009/2010 pandemic season (pandemic influenza), respectively. Estimated by using Cox proportional-hazards regression with follow-up between January 1, 2006, and December 31, 2013. Adjusted HRs also displayed graphically to the far right*

Fetal Death in the Third Trimester with Seasonal and Pandemic Influenza

		Number of birth records	Number of fetal deaths[1]	Crude HR (95% CI)	Adjusted HR (95% CI)[2]
	Total	414,972	1,040	—	—
Model 1	*Seasonal influenza*				
	No	408,334	1,023	1	1
	Yes	8,307	17	0.86 (0.53–1.39)	0.89 (0.55–1.43)
	Pandemic influenza				
	No	412,099	1,028	1	1
	Yes	3,559	12	1.42 (0.80–2.51)	1.44 (0.82–2.55)
Model 2	*Seasonal influenza*				
	No	408,334	1,023	1	1
	Yes, in the first trimester	3,027	7	0.93 (0.44–1.97)	0.97 (0.46–2.05)
	Yes, in the second or third trimester	5,280	10	0.82 (0.44–1.52)	0.84 (0.45–1.57)
	Pandemic influenza				
	No	412,099	1,028	1	1
	Yes, in the first trimester	1,394	8	2.28 (1.14–4.57)	2.34 (1.17–4.69)
	Yes, in the second or third trimester	2,165	4	0.81 (0.30–2.16)	0.81 (0.30–2.17)

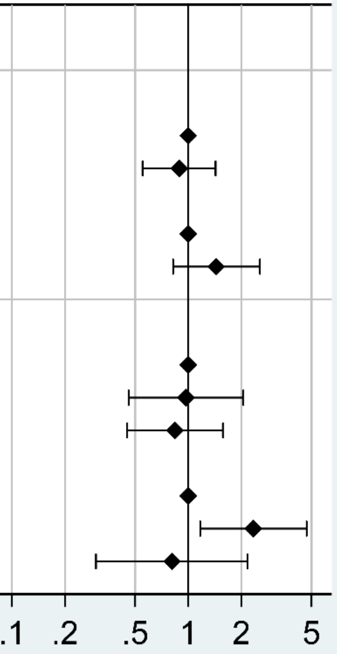

[1]Spontaneous abortions (miscarriages) and stillbirths.
[2]Ajustment for maternal age, maternal marital status, maternal parity, maternal history of fetal death, maternal chronic conditions (asthma, chronic hypertension, chronic renal disease, rheumatoid arthritis, heart disease, epilepsy, diabetes, or thyroid disease), maternal use of nutritional supplements before and/or during pregnancy, and maternal smoking at the beginning of pregnancy as baseline covariates and calendar season and maternal seasonal/pandemic influenza as time-dependent covariates.

Supplementary Fig. 2 *Hazard ratios (HRs) of fetal death in the third trimester, with associated 95% confidence intervals (CIs), between women with and without a diagnosis of influenza-like illness in pregnancy during regular influenza seasons (seasonal influenza) and with and without a diagnosis of influenza-like illness in pregnancy during the 2009/2010 pandemic season (pandemic influenza), respectively. Estimated by using Cox proportional-hazards regression with follow-up between January 1, 2006, and December 31, 2013. Adjusted HRs also displayed graphically to the far right*

Fetal Death after the First Trimester with Pandemic Influenza and Vaccination

		Number of birth records	Number of fetal deaths[1]	Crude HR (95% CI)	Adjusted HR (95% CI)[2]
	Total	113,135	451	—	—
Model 3	*Pandemic influenza*				
	No	111,493	424	1	1
	Yes	3,578	27	1.85 (1.25–2.74)	1.84 (1.23–2.73)
	Pandemic vaccination				
	No	108,441	363	1	1
	Yes	26,303	88	0.94 (0.74–1.20)	0.86 (0.66–1.12)
Model 4	*Pandemic influenza*				
	No	111,493	424	1	1
	Yes, in the first trimester	1,405	18	2.44 (1.52–3.91)	2.40 (1.49–3.87)
	Yes, in the second or third trimester	2,173	9	1.25 (0.64–2.42)	1.24 (0.64–2.41)
	Pandemic vaccination				
	No	108,441	363	1	1
	Yes, in the first trimester	2,465	12	0.88 (0.50–1.57)	0.86 (0.48–1.53)
	Yes, in the second or third trimester	23,838	76	0.95 (0.74–1.23)	0.86 (0.65–1.14)

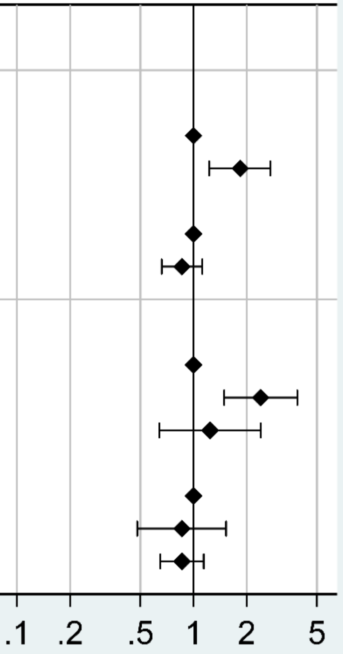

[1]Spontaneous abortions (miscarriages) and stillbirths.
[2]Ajustment for maternal age, maternal marital status, maternal parity, maternal history of fetal death, maternal chronic conditions (asthma, chronic hypertension, chronic renal disease, rheumatoid arthritis, heart disease, epilepsy, diabetes, or thyroid disease), maternal use of nutritional supplements before and/or during pregnancy, and maternal smoking at the beginning of pregnancy as baseline covariates and calendar season and maternal pandemic influenza/vaccination as time-dependent covariates.

Supplementary Fig. 3 *Hazard ratios (HRs) of fetal death in the second or third trimester, with associated 95% confidence intervals (CIs), between women with and without a diagnosis of influenza-like illness in pregnancy during the 2009/2010 pandemic season (pandemic influenza) and with and without vaccination against pandemic influenza virus in pregnancy (pandemic vaccination), respectively. Estimated by using Cox proportional-hazards regression with follow-up between May 15, 2009, and September 30, 2010. Adjusted HRs also displayed graphically to the far right*

Fetal Death in the Second Trimester with Pandemic Influenza and Vaccination

		Number of birth records	Number of fetal deaths[1]	Crude HR (95% CI)	Adjusted HR (95% CI)[2]
	Total	98,774	261	—	—
Model 3	*Pandemic influenza*				
	No	97,132	245	1	1
	Yes	2,869	16	2.24 (1.35–3.73)	2.09 (1.25–3.51)
	Pandemic vaccination				
	No	94,080	220	1	1
	Yes	17,414	41	1.14 (0.81–1.59)	0.98 (0.68–1.42)
Model 4	*Pandemic influenza*				
	No	97,132	245	1	1
	Yes, in the first trimester	1,405	11	2.52 (1.37–4.61)	2.35 (1.28–4.32)
	Yes, in the second trimester	1,464	5	1.81 (0.75–4.38)	1.67 (0.69–4.06)
	Pandemic vaccination				
	No	94,080	220	1	1
	Yes, in the first trimester	2,465	6	0.77 (0.34–1.73)	0.74 (0.33–1.66)
	Yes, in the second trimester	14,949	35	1.24 (0.87–1.78)	1.06 (0.70–1.61)

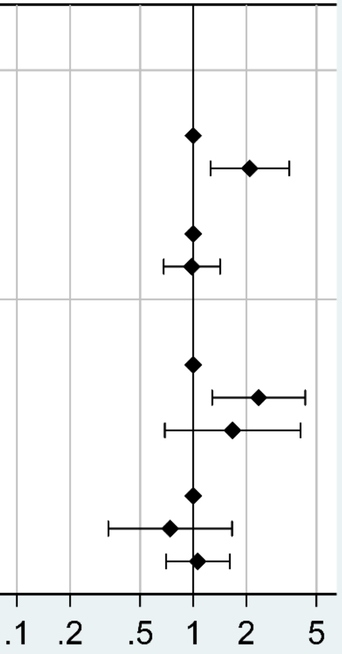

[1]Spontaneous abortions (miscarriages) and stillbirths.
[2]Ajustment for maternal age, maternal marital status, maternal parity, maternal history of fetal death, maternal chronic conditions (asthma, chronic hypertension, chronic renal disease, rheumatoid arthritis, heart disease, epilepsy, diabetes, or thyroid disease), maternal use of nutritional supplements before and/or during pregnancy, and maternal smoking at the beginning of pregnancy as baseline covariates and calendar season and maternal pandemic influenza/vaccination as time-dependent covariates.

Supplementary Fig. 4 *Hazard ratios (HRs) of fetal death in the second trimester, with associated 95% confidence intervals (CIs), between women with and without a diagnosis of influenza-like illness in pregnancy during the 2009/2010 pandemic season (pandemic influenza) and with and without vaccination against pandemic influenza virus in pregnancy (pandemic vaccination), respectively. Estimated by using Cox proportional-hazards regression with follow-up between May 15, 2009, and September 30, 2010. Adjusted HRs also displayed graphically to the far right*

Fetal Death in the Third Trimester with Pandemic Influenza and Vaccination

		Number of birth records	Number of fetal deaths[1]	Crude HR (95% CI)	Adjusted HR (95% CI)[2]
	Total	95,085	187	—	—
Model 3	*Pandemic influenza*				
	No	92,221	176	1	1
	Yes	3,542	11	1.50 (0.81–2.75)	1.49 (0.80–2.76)
	Pandemic vaccination				
	No	77,401	141	1	1
	Yes	26,230	46	0.79 (0.57–1.10)	0.69 (0.47–1.03)
Model 4	*Pandemic influenza*				
	No	92,221	176	1	1
	Yes, in the first trimester	1,377	7	2.37 (1.12–5.05)	2.26 (1.06–4.81)
	Yes, in the second or third trimester	2,165	4	0.91 (0.34–2.45)	0.92 (0.34–2.50)
	Pandemic vaccination				
	No	77,401	141	1	1
	Yes, in the first trimester	2,453	6	1.04 (0.46–2.36)	1.05 (0.46–2.39)
	Yes, in the second or third trimester	23,777	40	0.76 (0.54–1.08)	0.64 (0.42–0.96)

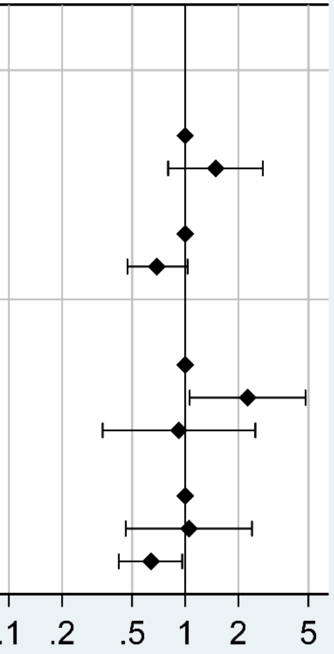

[1]Spontaneous abortions (miscarriages) and stillbirths.
[2]Ajustment for maternal age, maternal marital status, maternal parity, maternal history of fetal death, maternal chronic conditions (asthma, chronic hypertension, chronic renal disease, rheumatoid arthritis, heart disease, epilepsy, diabetes, or thyroid disease), maternal use of nutritional supplements before and/or during pregnancy, and maternal smoking at the beginning of pregnancy as baseline covariates and calendar season and maternal pandemic influenza/vaccination as time-dependent covariates.

Supplementary Fig. 5 *Hazard ratios (HRs) of fetal death in the third trimester, with associated 95% confidence intervals (CIs), between women with and without a diagnosis of influenza-like illness in pregnancy during the 2009/2010 pandemic season (pandemic influenza) and with and without vaccination against pandemic influenza virus in pregnancy (pandemic vaccination), respectively. Estimated by using Cox proportional-hazards regression with follow-up between May 15, 2009, and September 30, 2010. Adjusted HRs also displayed graphically to the far right*
